# Risk Prediction Score for Severe High Altitude Illness: A Cohort Study

**DOI:** 10.1371/journal.pone.0100642

**Published:** 2014-07-28

**Authors:** Florence Canouï-Poitrine, Kalaivani Veerabudun, Philippe Larmignat, Murielle Letournel, Sylvie Bastuji-Garin, Jean-Paul Richalet

**Affiliations:** 1 Université Paris Est, Faculté de Médecine, EA 4393, Laboratoire d'Investigation Clinique, Créteil, France; 2 AP-HP, Groupe Henri Mondor-Albert Chenevier, Service de Santé Publique, Créteil, France; 3 AP-HP, Hôpital Avicenne, Service d'Anesthésie et Réanimation, Bobigny, France; 4 Université Paris 13, Sorbonne Paris Cité, EA2363 « Réponses cellulaires et fonctionnelles à l'hypoxie », U.F.R. SMBH, Bobigny, France; 5 AP-HP, Hôpital Avicenne, Service de Physiologie, Explorations Fonctionnelles et Médecine du Sport, Bobigny, France; University Medical Center Freiburg, Germany

## Abstract

**Background:**

Risk prediction of acute mountain sickness, high altitude (HA) pulmonary or cerebral edema is currently based on clinical assessment. Our objective was to develop a risk prediction score of Severe High Altitude Illness (SHAI) combining clinical and physiological factors. Study population was 1017 sea-level subjects who performed a hypoxia exercise test before a stay at HA. The outcome was the occurrence of SHAI during HA exposure. Two scores were built, according to the presence (PRE, n = 537) or absence (ABS, n = 480) of previous experience at HA, using multivariate logistic regression. Calibration was evaluated by Hosmer-Lemeshow chisquare test and discrimination by Area Under ROC Curve (AUC) and Net Reclassification Index (NRI).

**Results:**

The score was a linear combination of history of SHAI, ventilatory and cardiac response to hypoxia at exercise, speed of ascent, desaturation during hypoxic exercise, history of migraine, geographical location, female sex, age under 46 and regular physical activity. In the PRE/ABS groups, the score ranged from 0 to 12/10, a cut-off of 5/5.5 gave a sensitivity of 87%/87% and a specificity of 82%/73%. Adding physiological variables via the hypoxic exercise test improved the discrimination ability of the models: AUC increased by 7% to 0.91 (95%CI: 0.87–0.93) and 17% to 0.89 (95%CI: 0.85–0.91), NRI was 30% and 54% in the PRE and ABS groups respectively. A score computed with ten clinical, environmental and physiological factors accurately predicted the risk of SHAI in a large cohort of sea-level residents visiting HA regions.

## Introduction

An increased number of sea-level residents visit areas above 4000 m of altitude for leisure, sport or work. They may suffer from severe acute mountain sickness, high altitude pulmonary or cerebral edema, especially when they are not yet acclimatized to the hypoxic environment [1]. The above clinical outcomes have been regrouped in a clinical entity called Severe High Altitude Illness (SHAI).

Recently, we assessed several clinical, environmental and physiological independent risk factors of SHAI in a cohort of 1326 sea-level residents who came to an outpatient mountain medicine consultation and underwent a hypoxic exercise test before a stay at altitude above 4000 m [2]. The main risk factors evidenced were previous history of SHAI, low ventilatory and cardiac response to hypoxia at exercise, speed of ascent above 400 m per day in the acclimatization period, high desaturation during exercise in hypoxia, history of migraine, geographical location (Aconcagua, Mont-Blanc, Ladakh), female gender, regular endurance training and age under 46 years old. Some of these factors such as history of SHAI or migraine were already mentioned in the literature [1], [3]–[6]. However this study was the first to demonstrate that adding physiological measurements to clinical evaluation greatly improved the discrimination between susceptible and non-susceptible subjects [2]. As 45% of the subjects coming to the outpatient consultation had no previous experience of sojourn at high altitude, one of the main risk factor (history of SHAI) could not be taken into account in the risk prediction.

The main aim of the present study was to develop and internally validate, in a large cohort of sea-level residents visiting high-altitude regions, a risk prediction score for SHAI occurrence including clinical and physiological factors. The secondary endpoint is to assess and quantify the benefit of performing a hypoxia exercise test to predict the risk of SHAI, especially in subjects without previous experience at high altitude. This score may be useful to detect highly susceptible subjects and improve the prevention of SHAI for newcomers at high altitude. This is a clear added value to our previous work where no prediction score was proposed. Moreover, we offer here a decisional tree for validation in other centers interested in mountain medicine.

## Methods

### Study design and data collection

A prospective open cohort study involved all subjects who came to the outpatient altitude medicine consultation at Avicenne Hospital (Bobigny, France) between January 1^st^, 1992 and December 31, 2011 before a stay of at least 3 days above 4000 m with an overnight sleeping above 3500 m. From the previous study [2], 102 subjects were added (10% of the population) to perform the score calculation. The present study is based on a 20-yr experience of evaluating persons before a stay at high altitude. Individual informed consent was collected. For this non-interventional study, no Ethics committee approval was needed according to French law.

For the subjects who came several times during the study period, only the first visit was included in the analysis. Subjects were given a questionnaire based on Hackett's score [7] thus allowing the definition of the clinical outcome (suffering or not from SHAI). None of the subjects spent more than 4 days above 3000 m in the preceding 2 months. Subjects who sent back the questionnaire and did not take acetazolamide or other medication used for altitude sickness (PDE5 inhibitors, calcium blockers or dexamethasone) during the stay at high altitude were finally included in the study.

#### Baseline examination

Subjects were questioned about their socio-demographic characteristics, personal and familial medical history, treatments, objective of the stay (tourism, mountaineering, trekking or work, precise geographical location), previous history of SHAI, recent stay at high altitude, previous history of migraine (criteria defined by the International Headache Society) [8], regular endurance training activity (at least 40 minutes, 3 times a week) as previously defined in detail [2]. Each subject performed a hypoxic exercise test (inspired fraction of O_2_: 0.115, exercise intensity of approximately 30% of maximal aerobic power that allowed the calculation of three main physiological parameters: desaturation induced by hypoxia at exercise, ventilatory response to hypoxia at exercise, and cardiac response to hypoxia at exercise [2], [9]–[11]. These last two parameters are calculated from the ratio of increased ventilation or heart rate over the decrease in arterial oxygen saturation induced by hypoxia during exercise. They are an indirect measurement of the chemosensitivity to a hypoxic challenge [2]. Data were collected from the patient's medical chart containing the clinical observations and the physiological measurements made by the physician who examined the patient at the consultation. Patient gave authorization for using his/her data and data were anonymized before any statistical treatment. The study was declared to French National data protection organization (Commission nationale de l'informatique et des libertés (CNIL)/registration number 1740068) and data collection and informatics treatments followed the principles of the CNIL.

#### Follow-up and clinical outcome

Outcome was the occurrence of SHAI. Each subject was asked to fill out a daily questionnaire based on Hackett's score of severe AMS and symptoms of HAPE or HACE during their future stay at high altitude and send it back upon returning home [7]. Moderate AMS was defined by a Hackett's score <6, without significant impact on planned activity. Severe AMS was defined by a Hackett's score ≥6, with incapacitating symptoms leading to a stop or a significant reduction of planned activity. HAPE was defined by the presence of clinical signs of respiratory distress (dyspnea, cyanosis, rales), confirmed by a thorax X-Ray upon descent to low altitude. HACE was defined by clinical signs of neurological deficit (ataxia, mental confusion). The diagnosis of HAPE or HACE was always confirmed by an expert, either on the spot where the disorder occurred or later on when hospitalized. Subjects were asked to note any medication taken during their stay, especially acetazolamide, widely used among trekkers for the prevention of AMS [12]. From the questionnaire, subjects were classified as no or moderate AMS, severe AMS, HAPE or HACE. Severe AMS, HAPE and HACE were pooled as SHAI and considered as being intolerant to high altitude. Information about the ascent characteristics (daily speed of ascent, altitude reached) was collected in the follow-up questionnaire. This questionnaire is part of the usual care as it is systematically given to all patients coming to the consultation in order to have a follow-up in case of health problems during the stay at high altitude.

### Statistical analysis

Qualitative variables were expressed in N (%), quantitative variables in mean (standard deviation SD) or median (25^th^-75^th^ percentiles as appropriate).

History of SHAI is a major risk factor of SHAI [1], [2]. However, it can be determined only in subjects with previous exposure to high altitude. Therefore the entire analysis was stratified according to previous history (or not) of high altitude sojourn. The baseline characteristics of subjects were compared according to their previous history (or not) of sojourn at high altitude using Student t-test or Wilcoxon-Mann-Whitney and the Pearson chi square-test or Fisher's exact test, as appropriate. Due to deviation from linearity assumption, physiological variables were expressed in dummy variables defined by tertiles in the group of subjects without SHAI.

Two multivariate models were built for each group (previous sojourn or not), one with the classical risk factors and one with supplemental adjustment for physiological variables. The variables entered in the multivariate model were those previously identified: history of SHAI (only in the group with previous high altitude sojourn), planned rapid ascent, history of migraine, geographical location, age, sex, physical activity for the classical risk factors and hypoxic ventilatory response, hypoxic cardiac response and desaturation in hypoxia at exercise for physiological variables [2]. Two-by-two analyses were performed to assess potential interactions and confounding by fitting. We examined interactions between these variables, and between these variables and the presence of previous high altitude sojourn.

We developed risk prediction scores using established methods in both groups [13]–[15]. We used a multivariate logistic regression model to estimate the β coefficients for each risk factor. Variables were retained in the final multivariate model if they had an odds ratio (OR) of more than 1.2 (for binary variables) [14] and p<0.20. We internally validated the scores by using bootstrap resampling with 1000 replications of both groups to estimate β’ shrinkage coefficients [16]. We used the median bootstrapped regression coefficients rounded to the nearest half point as weights, which we combined with the baseline logit function to derive risk prediction scores. We assessed calibration and discrimination of the multivariate models and of the scores in the two groups. Calibration was evaluated by computing the Hosmer-Lemeshow chi-square statistic and discrimination by the C-statistic (Area Under ROC Curve, AUC) with 95% confidence intervals: the usual thresholds were considered: ≥0.7 =  good discrimination; ≥0.8: very good and ≥0.9 =  excellent. Discrimination was also quantified by calculating the Net Reclassification Improvement index (NRI): NRI indicates the proportion of patients correctly classified (in the group who will and the group who will not develop SHAI) when adding physiological markers to the clinical model [17].

We calculated the sensitivity, specificity, predictive values and proportion of correctly classified subjects for each value of the risk prediction score to determine thresholds.

All tests were two-tailed. No imputation of missing data was made. Data were analyzed using STATA software version 12 (Stata Inc., College Station, TX). Reporting of the present study was done using STrengthening the Reporting of OBservational studies in Epidemiology (STROBE) guidelines.

## Results

### Baseline characteristics

**Figure 1:**
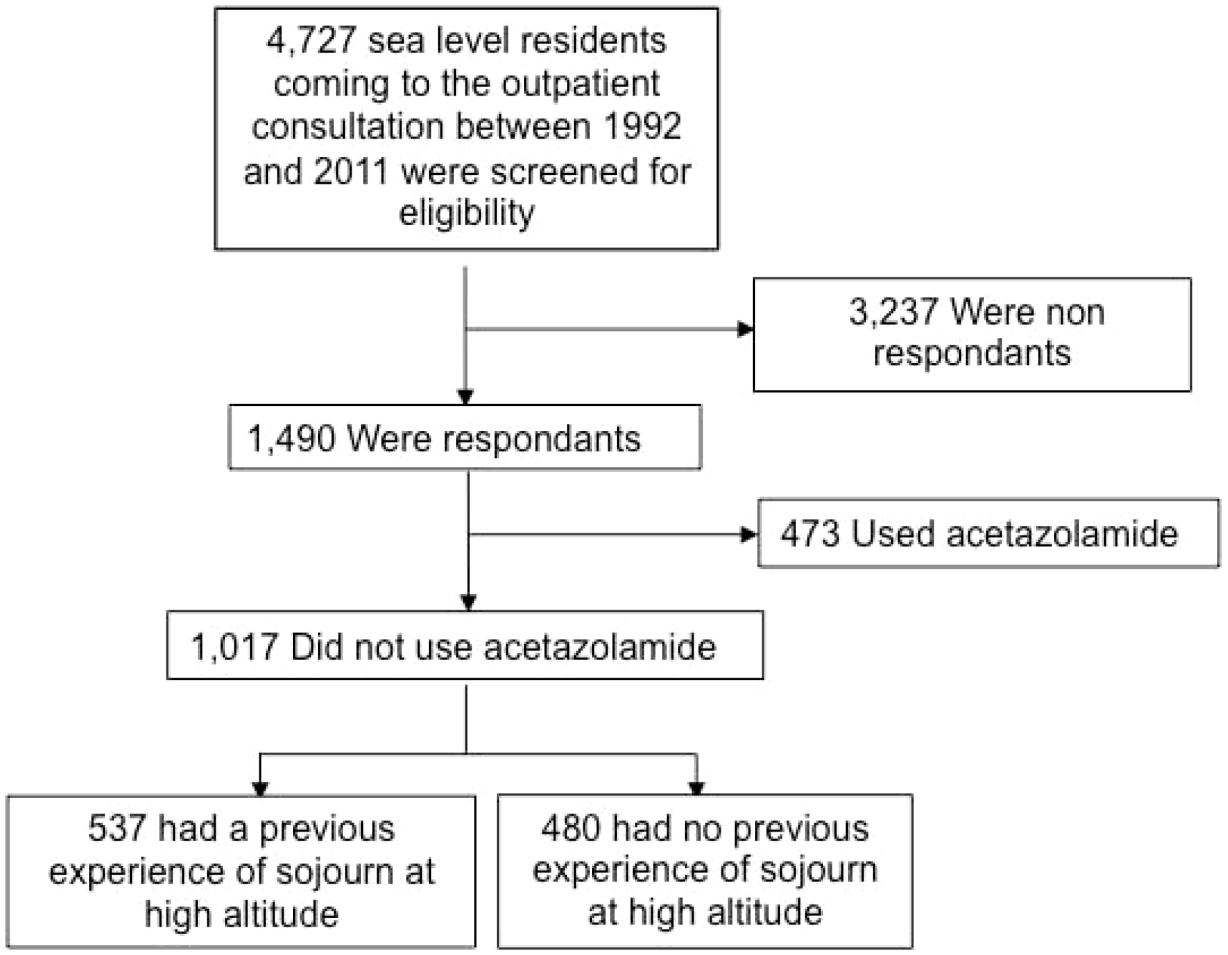
Flow chart of the study.

Overall, 4727 subjects came for the first time to the outpatient altitude medicine consultation at Avicenne Hospital between January 1^st^, 1992 and December 31, 2011. After exclusion of non-respondents and subjects who took acetazolamide, 1017 subjects were included in the study (Figure 1). Baseline characteristics of groups with and without previous high altitude sojourn are shown in Table 1. Subjects with no previous sojourn at high altitude were younger, more frequently women, had higher blood pressure, lower planned rapid ascent, less frequent special geographical locations and regular endurance physical activity. No difference was found regarding physiological variables.

**Table 1:** Characteristics of the patients according to history of previous high altitude sojourn.

	Previous high altitude sojourn (N = 537)	No previous high altitude sojourn (N = 480)	P*
Age - yr	45.8 (13.5)	42.6 (15.1)	<0.0004
Female sex - no. (%)	164 (31.4)	214 (45.1)	<0.001
Current smokers – no. (%)	55 (10.5)	49 (10.3)	0.92
Body Mass Index – kg/m^2^ ≥25 kg/m^2^	93 (17.8)	95 (20.0)	0.37
Systolic Blood Pressure - mmHg	132.3 (14.1)	128.4 (14.1)	<0.001
History of Severe High Altitude Illness – no. (%)	126 (24.1)	-	-
History of migraine – no. (%)	71 (13.6)	51 (10.8)	0.17
Rapid ascent (>400 m/day) – no. (%)	157 (30.54)	112 (24.0)	0.023
Geographical location (Aconcagua, Mont Blanc or Ladakh) - no. (%)	121 (23.1)	56 (11.8)	<0.001
Regular endurance physical activity – no. (%)	216 (41.4)	108 (22.7)	<0.001
Hypoxic ventilatory response at exercise - l/min/kg <0.68	225 (44.0)	207 (44.2)	
(0.68–0.94)	152 (29.7)	136 (29.1)	0.98
≥0.94	135 (26.4)	125 (26.7)	
Hypoxic cardiac response at exercise - b/min/% <0.72	193 (37.6)	160 (34.1)	
(0.72–0.95)	171 (33.3)	159 (33.9)	0.46
≥0.95	149 (29.1)	150 (32.0)	
Desaturation at exercise in hypoxia - % <19	149 (28.5)	147 (31.1)	
(19–24)	178 (34.1)	155 (32.8)	0.67
≥24%	195 (37.4)	170 (36.0)	

Data are expressed as n (%) or means (SD); * Pearson chisquare or Student t-test as appropriate

### Subjects with previous sojourn at high altitude

The multivariate model included history of SHAI, rapid ascent, geographical location, female sex and regular endurance physical activity had a very good discrimination (AUC = 0.84) and an adequate calibration (p-value Hosmer-Lemeshow chisquare = 0.87) (Table 2). When adding physiological variables, the discrimination significantly increased by 7.5% up to an excellent discrimination (>0.90). The multivariate physio-clinical model showed an adequate calibration (Table 2). A 30% NRI indicates that 30% of subjects were correctly reclassified when adding the physiological variables into the predictive model (toward lower risk category for subjects who will not developed SHAI, and toward high risk category for subjects who will developed SHAI). Desaturation was not included in the physio-clinical model due to close correlation with history of SHAI leading to overadjustement and default of calibration. We found no significant interaction.

**Table 2:** Adjusted Odds Ratios (95% CI) for clinical and physio-clinical multivariate model and scoring system in subjects **with** previous high-altitude sojourn (n = 501, 36 missing).

	Clinical model		Physio-clinical model			
Variables	Odds Ratio (95% CI) *	P Value†	Odds Ratio (95% CI) *	P value†	β’ regression coefficient‡	Points ¶
History of Severe High Altitude Illness	12.35 (7.24–21.08)	<0.001	12.89 (6.78–24.49)	<0.001	2.58	2.5
Rapid ascent (> 400 m/night)	4.69 (2.79–7.90)	<0.001	5.89 (3.19–10.87)	<0.001	1.84	2
History of migraine	2.21 (1.15–4.24)	0.017	4.29 (1.93–9.54)	<0.001	1.27	1.5
Geographical location (Aconcagua, Mt Blanc, Ladakh)	2.7 (1.47–4.88)	0.001	2.43 (1.28–4.61)	0.006	0.79	1
Age < 46 years	1.62 (1.00–2.63	0.05	1.82 (1.00–3.29)	0.049	0.48	0.5
Female sex	1.60 (0.96–2.67)	0.073	1.38 (0.75–2.54)	0.30	0.29	0
Regular endurance physical activity	1.9 (1.09–3.19)	0.53	1.46 (0.80–2.65)	0.12	0.45	0.5
Hypoxic ventilatory response at exercise (l/min/kg)	-	-				
low < 0.68			20.59 (6.76–62.7)	<0.001	3.07	3
moderate (0.68–0.94)	-	-	3.41 (1.10–10.59)	0.034	1.18	1
high ≥0.94	-	-	ref	ref	ref	0
Hypoxic cardiac response at exercise (b/min/%)	-	-				
low <0.72			2.41 (1.16–5.03)	0.019	0.97	1
moderate (0.72–0.95)	-	-	0.94 (0.44–2.01)	0.89	0.07	0
high ≥0.95	-	-	ref	ref	ref	0
C-statistic (Area Under ROC Curve) (CI 95%)‡	0.84 (0.78–0.88)	-	0.91 (0.87–0.93)			
Calibration: Hosmer-Lemeshow chisquare	3.81 (p = 0.87)	-	4.52 (p = 0.81)			
Net Reclassification Index ¥	-	-	30% (p<0.001)			

*Adjusted odds ratio from multivariate logistic regression adjusted for all variables listed in the column; † Wald test; ‡ Estimations obtained after 1000 resampling; ¶ β’ Coefficient rounded to the near half integer; ¥ Net Reclassification Index indicates the proportion of patients correctly classified (in the group who will and the group who will not develop SHAI when adding physiological variables to the clinical model.

Bootstrapped multivariate regression β’ coefficients were almost similar to those of original multivariate regression model [2]. Points rounded to the nearest half point were assigned to the bootstrapped β’ coefficients (Table 2). The score was then computed by means of a linear combination of the rounded coefficients as follows:

SHAI risk prediction score for subjects with previous exposure to high altitude  = 

2.5* (History of SHAI)

+2* (ascent > 400 m/day)

+1.5* (history of migraine)

+1*(Geographical location  =  Aconcagua, Mont Blanc or Ladakh)

+0.5* (age<46 years)

+0.5* (regular physical activity)

+3* (hypoxic ventilatory response at exercise <0.68 L/min/kg) or 1*(0.68< hypoxic ventilatory response at exercise <0.94 L/min/kg)

+1* (hypoxic cardiac response at exercise <0.72 b/min/%).

**Figure 2:**
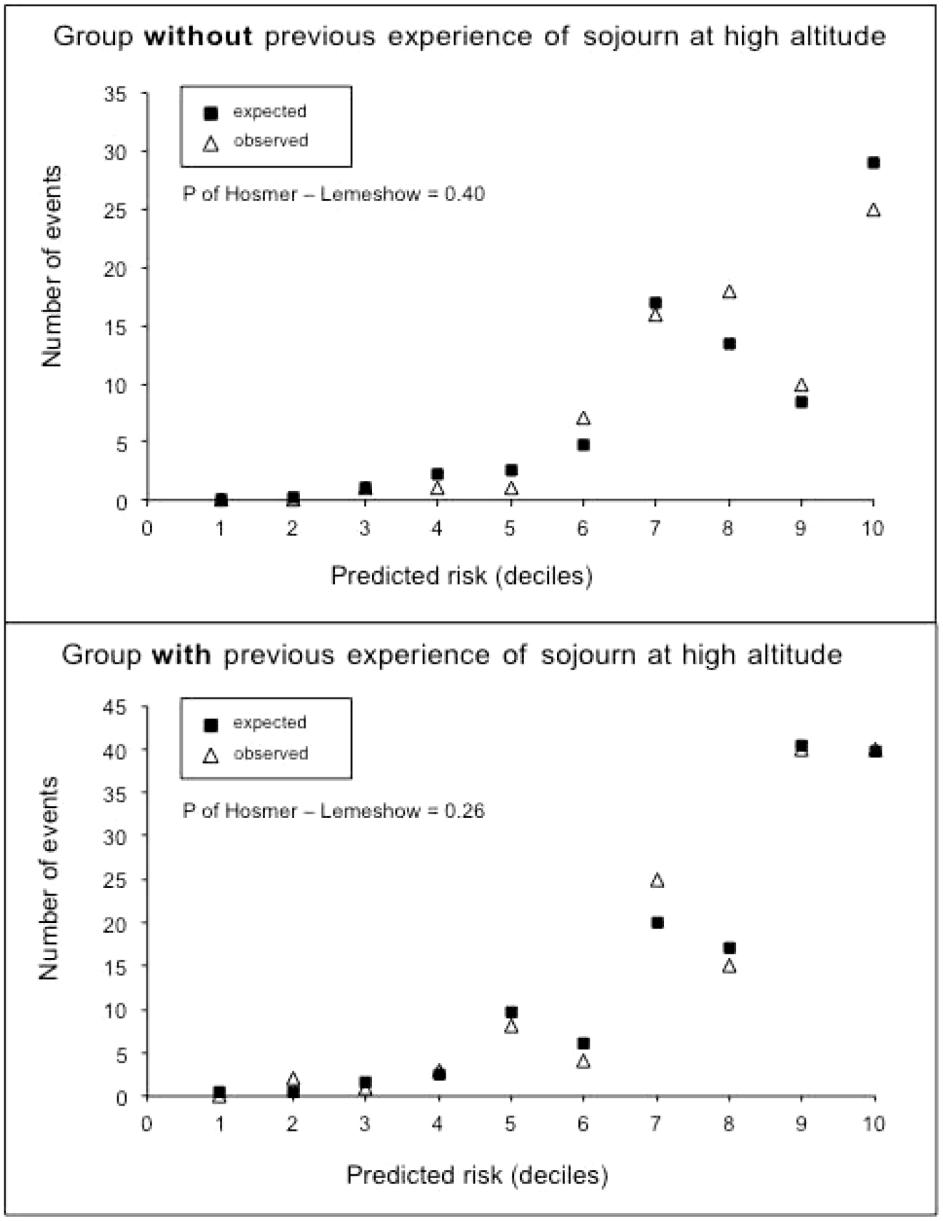
Calibration of the risk prediction score for Severe High Altitude Illness in the group without previous experience of sojourn at high altitude (upper panel, n = 480) and with previous experience of sojourn at high altitude (lower panel, n = 537).

**Figure 3:**
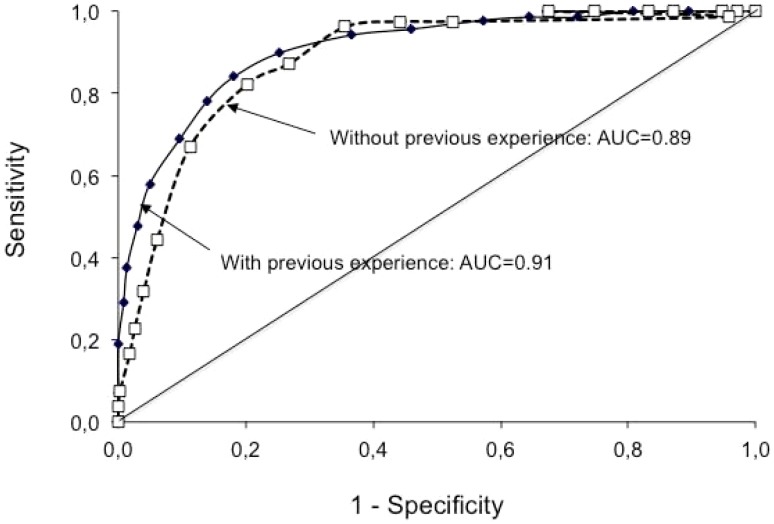
ROC curve of the risk prediction score for Severe High Altitude Illness in the groups without (n = 480) and with (n = 537) previous experience of sojourn at high altitude.

The calibration of the score was adequate (Figure 2, lower panel) and the discrimination was very good (Figure 3). Median SHAI risk prediction score was 3 (1.5–4.5) in patients free of SHAI and 6.5 (5.5–8) in patients with SHAI. A cut-off of 5 gave a sensitivity of 87%, a specificity of 81.8%, a positive likelihood ratio of 4.8 and negative likelihood ratio of 0.16.

### Subjects without previous sojourn at high altitude

The multivariate model included history of SHAI, rapid ascent, geographical location, female sex and regular endurance physical activity had adequate calibration (p-value Hosmer-Lemeshow chisquare = 0.91) and good discrimination (AUC = 0.72) (Table 3). When adding physiological variables, the discrimination significantly increased by 17% up to a very good discrimination (AUC>0.80). The multivariate physio-clinical model showed an adequate calibration (p-value Hosmer-Lemeshow chisquare = 0.36) (Table 3). A 54% NRI indicates that 54% of subjects were correctly reclassified when adding the physiological variables into the predictive model. We found no significant interaction.

**Table 3:** Adjusted Odds Ratios (95% CI) for clinical and physio-clinical multivariate model and scoring system in subjects **without** previous high-altitude sojourn (n = 457, 23 missing).

	Clinical model		Physio-clinical model			
Variables	Odds Ratio (95% CI) *	P Value†	Odds Ratio (95% CI) *	P Value†	β’ regression coefficient‡	Points ¶
History of Severe High Altitude Illness	-	-	-	-	-	-
Rapid ascent (>400 m/night)	4.26 (2.54–7.16)	<0.001	7.26 (3.70–14.22)	<0.001	2.09	2
History of migraine	1.18 (0.55–2.55)	0.67	1.31 (0.51–3.33)	0.57	0.26	0
Geographical location (Aconcagua, Mt Blanc, Ladakh)	1.31 (0.63–2.75)	0.47	1.87 (0.76–4.61)	0.18	0.63	0.5
Age < 46 years	1.44 (0.84–2.46)	0.18	1.47 (0.77–2.79)	0.24	0.42	0
Female sex	1.51 (0.90–2.52)	0.12	1.73 (0.94–3.18)	0.08	0.6	0.5
Regular endurance physical activity	1.68 (0.96–2.94)	0.07	2.00 (1.00–3.99)	0.049	0.75	1
Hypoxic ventilatory response at exercise (l/min/kg)	-	-		<0.001		
low <0.68			17.3 (3.62 –82.47)		3.12	3
moderate (0.68–0.94)	-	-	3.28 (0.66–16.4)		1.30	1
high ≥0.94	-	-	ref		ref	0
Hypoxic cardiac response at exercise (b/min/%)	-	-		0.12		
low <0.72			2.19 (0.94–5.06)		0.85	1
moderate (0.72–0.95)	-	-	2.22 (0.97–5.11)		0.85	1
high ≥0.95	-	-	ref		ref	0
Desaturation at exercise in hypoxia - %	-	-		<0.001		
low <19			ref		ref	0
moderate (19–24)	-	-	3.29 (0.86–12.31)		1.23	1
high ≥24		-	9.03 (2.48–32.89)		2.3	2
C-statistic (Area Under ROC Curve) (CI 95%)‡	0.72 (0.66–0.78)		0.89 (0.85–0.91)			
Calibration: Hosmer-Lemeshow chisquare	2.68 (p = 0.91)		8.79 (p = 0.36)			
Net Reclassification Index ¥			54% (p<0.001)			

*Adjusted odds ratio from multivariate logistic regression adjusted for all variables listed in the column; † Wald test; ‡ Estimations obtained after 1000 resampling; ¶ β’ Coefficient rounded to the near half integer; ¥ Net Reclassification Index indicates the proportion of patients correctly classified (in the group who will and the group who will not develop SHAI when adding physiological variables to the clinical model.

The score was computed as for the previous group, but with different coefficients (Table 3):

SHAI risk prediction score for subjects without previous exposure to high altitude  = 

2* (ascent > 400 m/day)

+0.5* (Geographical location  =  Aconcagua, Mont Blanc or Ladakh)

+0.5* (female sex)

+1* (regular physical activity)

+3* (hypoxic ventilatory response at exercise <0.68 L/min/kg) or 1* (0.68< hypoxic ventilatory response at exercise < 0.94 L/min/kg)

+1* (hypoxic cardiac response at exercise < 0.95 b/min/%)

+2* (desaturation at exercice in hypoxia ≥24%) or 1* (19%<desaturation at exercice in hypoxia <24%).

The calibration of the score was adequate (Figure 2, upper panel) and the discrimination was very good (Figure 3). Median SHAI risk prediction score was 4 (2–5.5) in patients free of SHAI and 6.5 (6–7.5) in patients with SHAI. A cut-off of 5.5 gave a sensitivity of 87.3%, a specificity of 73.1%, a positive likelihood ratio of 3.2 and a negative likelihood ratio of 0.17.

We included female sex and migraine in the two multivariate models (for the groups with and without previous high-altitude sojourn). However, in the multivariate analysis after adjustment for others confounders, the variable female sex in the group with previous experience and the variable migraine in the group without remained not significant leading to a nul weight in the final score.

## Discussion

This is the first study proposing a risk prediction scoring system for SHAI based on a large cohort of sea-level residents visiting high altitude regions. Ten clinical, environmental and physiological variables were used to compute the risk prediction scoring, with a different weight applied to the 10 items according to presence or absence of previous experience at high altitude. It ranged from 0 to 10 points in subjects without previous experience and 0 to 12 in those with. The obtained scoring systems had very good to excellent discrimination ability and adequate calibration. The two groups showed some different characteristics (Table 1): as it might be expected, subjects with previous experience at high altitude were older, more trained, more men, had slightly higher blood pressure and went to higher risk locations. These differences justify the stratification made before performing the multivariate logistic regression. We chose to develop two separate multivariate models leading to two prediction scores, in the group of subjects with previous exposure and in the group of subjects without. Indeed, even if the two multivariate models are very close, some predictors differ, precluding the possibility of using one group as development and the other one as validation datasets. In particular, the weight of history of SHAI (available only in the group with previous exposure to high altitude) modifies the weight of the other variables of the model and therefore modifies the final score.

Previous episodes of SHAI, history of migraine and high speed of ascent are risk factors already mentioned in several studies [4], [5], [18]. Adding physiological variables linked to the tolerance to hypoxia during moderate exercise increases the accuracy of the prediction and allows a mechanistic approach [2], [18]–[20]. In subjects with previous experience of high altitude exposure, adding physiological variables allowed a reclassification of 30% of the subjects, while in altitude naïve subjects, 54% of the subjects were reclassified. This clearly demonstrates the benefit of performing a hypoxia exercise test in subjects before a sojourn at high altitude, especially when no information is available about their susceptibility to SHAI, which was the case in almost half of our consultants. The fact that regular endurance training is not a protecting factor for SHAI is important to mention; in fact it is rather detrimental, due to a more severe desaturation during exercise in this population compared to sedentary subjects [21]. Including the geographical location in the prediction score allows a personalization of the prevention process. However, as Mont-Blanc is mainly climbed by Europeans, it might be considered as a surrogate for any mountain around 4800 m high, the ascent of which is done without prior acclimatization. It is important to mention that this higher risk is adjusted for speed of ascent, i.e the risk of developing AMS in Mt Blanc, Ladakh or Aconcagua is still higher than other locations independently from the speed of ascent. So that there is something more we do not explain, or because people who climb Mont-Blanc come from the big cities and do not take time to acclimatize before climbing the mountain. No subject was acclimatized (no recent stay above 3000 m) when they travelled to high altitude, so that pre-acclimatization, which is a well known factor reducing the risk of SHAI was not taken into in account in our prediction model.

The strength of the study was the prospective design, the use of validated criterion to assess SHAI, and the large population with systematic measurement of physiological response to hypoxia at baseline. To develop and validate the score, we used a rigorous procedure for involving internal validation (bootstrap of the β regression coefficient). Shrinkage of the regression coefficients aims to correct for over-optimism in the model and may help to make models more transportable [16]. We used bootstrap resampling with 1000 replications of both groups and not split-sample analyses as it has been shown by Steyerberg et al that the latter methods gave overly pessimistic estimates whereas the former provided stable estimates with low bias [16].

Other tests have been proposed to assess, for example, the specific individual susceptibility to HAPE by measuring the pulmonary vascular reactivity to hypoxia and/or exercise [22]. More usual tests such as ECG or pulmonary function tests have failed to demonstrate any prediction power [1]. Finally, our approach, here, is not mechanistic, looking at the precise physiopathological causes of HAPE or HACE, although we address the basic common reason of all severe manifestations, i.e. severe hypoxemia, whatever the following effects on the lungs or on the brain. Our approach is very practical for a physician in front of a person who wants to know his own level of risk. The common trait of all SHAI manifestations is that the subject has to stop his trip and take urgent measures (such as descent, re-oxygenation, drugs…).

The reported results should be interpreted with some limitations in mind. The response rate was low, leading to a possible selection bias, although no major difference was found in the clinical and physiological characteristics between responders and non-responders (results not shown). However, we cannot discard the possibility that non-respondents had a successful stay at altitude and perhaps felt less compelled to return the questionnaire. Classification of SHAI was based on a self-evaluation without immediate medical control leading to a possible classification bias. However an expert using a validated score made the adjudication. Similarly, information concerning speed of ascent and history of migraine was self-declarative and subject to caution. We used the Hackett's score since when the study was started, in 1991, the Lake Louise score was not yet in use for epidemiological studies of AMS. Besides, the Hackett's score has been highly correlated to the Lake Louise score and the Environmental Symptoms Questionnaire for the clinical evaluation of AMS [23]. Although we included a long list of potential predictor variables and established which factors remain independent after adjustment and their relative importance [2], a residual confounding bias may persist.

Finally, this study was conducted from a single outpatient altitude medicine consultation and its transportability has to be evaluated in other populations, by means of an external validation. However, the recruitment of this consultation comes from all regions of France and surrounding French-speaking countries.

### Practical considerations for physicians

For a given set of variables entered in the score, a physician can compute the probability of developing SHAI during a stay at high altitude. This probability depends on intrinsic factors such as sex, history of migraine, physiological response to hypoxia, endurance training status which are fixed for a given individual, but also on extrinsic factors such as the geographical location of the destination or the speed of altitude ascent that can be modified by the individual, following the advice of the physician. There could be a certain degree of uncertainty concerning one of the three most important factors imbedded in the score, i.e. the history of SHAI since about 44% of the population coming to the consultation has never been at high altitude beforehand. To avoid this bias, the physician can compute one of the two versions of the prediction score considering the presence or absence of previous exposure to high altitude. Finally, for a patient with a score above the cut-off value of 4.5 or 5.5, the physician may propose further preventive measures such as alternative routes or preventive medication with acetazolamide which has been shown to reduce by almost 50% the relative risk of SHAI at the dose of 250 mg/day, starting the day before reaching 3,000 m of altitude [2]. In any case, this consultation will be the occasion to inform all the patients about the guidelines of prevention and treatment of severe high-altitude as well as cold-related diseases [1], [24]. Based on objective measurements, this score may be a useful tool for physicians practicing High Altitude medicine consultations, allowing a better prevention of the risk of severe medical problems in newcomers to high altitude. More effective prevention could therefore reduce the risk of costly evacuations from remote regions of the world.

In conclusion, we developed a scoring system based on 10 clinical, environmental and physiological factors that accurately predicted the SHAI risk in sea-level residents visiting high altitude regions. The use of a hypoxia exercise test greatly improved the prediction, especially in subjects with no previous experience of high altitude sojourn. The three most robust risk factors of SHAI are previous history of SHAI, excessive speed of ascent and hypoxic ventilatory response at exercise. A validation in an independent cohort undertaken by an independent team is needed to confirm its transportability. These findings may be useful to clinicians for reducing the risk of SHAI.
